# Boosting Adaptive Immunity: A New Role for PAFR Antagonists

**DOI:** 10.1038/srep39146

**Published:** 2016-12-14

**Authors:** Marianna M. Koga, Bruna Bizzarro, Anderson Sá-Nunes, Francisco J. Rios, Sonia Jancar

**Affiliations:** 1 Department of Immunology, Institute of Biomedical Sciences, University of São Paulo, São Paulo, Brazil; 2 Institute of Cardiovascular and Medical Sciences, British Heart Foundation Glasgow Cardiovascular Research Centre, University of Glasgow, Glasgow, United Kingdom

## Abstract

We have previously shown that the Platelet-Activating Factor Receptor (PAFR) engagement in murine macrophages and dendritic cells (DCs) promotes a tolerogenic phenotype reversed by PAFR-antagonists treatment *in vitro*. Here, we investigated whether a PAFR antagonist would modulate the immune response *in vivo.* Mice were subcutaneously injected with OVA or OVA with PAFR-antagonist WEB2170 on days 0 and 7. On day 14, OVA–specific IgG2a and IgG1 were measured in the serum. The presence of WEB2170 during immunization significantly increased IgG2a without affecting IgG1 levels. When WEB2170 was added to OVA in complete Freund’s adjuvant, enhanced IgG2a but not IgG1 production was also observed, and CD4+ FoxP3+ T cell frequency in the spleen was reduced compared to mice immunized without the antagonist. Similar results were observed in PAFR-deficient mice, along with increased Tbet mRNA expression in the spleen. Additionally, bone marrow-derived DCs loaded with OVA were transferred into naïve mice and their splenocytes were co-cultured with fresh OVA-loaded DCs. CD4^+^ T cell proliferation was higher in the group transferred with DCs treated with the PAFR-antagonist. We propose that the activation of PAFR by ligands present in the site of immunization is able to fine-tune the adaptive immune response.

The biologically active lipid known as the Platelet-Activating factor (PAF; 1-0-alkyl-2-acetyl-sn-glycero-3-phosphocholine) is produced from membrane phospholipids through enzymatic hydrolysis catalyzed by phospholipase A2. PAF is a potent mediator of inflammatory events such as increased vascular permeability, smooth muscle contraction, inflammatory cells migration, and wound healing, among other effects^1^. The receptor for PAF (PAFR), is a G protein-coupled receptor that was cloned in 1991 by Honda *et al*.^2^, and is linked to different subunits of G proteins, depending on its localization: the plasma membrane receptor is linked to Gα_q_ protein, whereas the nuclear PAFR seems to be linked to G_i/o_ protein^3^. Additionally, it has been proposed that the plasma membrane PAFR would induce immediate PAF responses and PAF synthesis, which would subsequently act on nuclear receptors in order to induce the synthesis of cytokines and enzymes that are responsible for prostanoid and nitric oxide (NO) synthesis^4^,^5^. A series of PAFR antagonists have been described, and they are important tools used to understand the role of PAF in physiological and pathological conditions^6^.

PAF has also been shown to induce striking functional changes in macrophages. Analysis from our laboratory showed that in human and murine macrophages, the engagement of PAFR by oxidized low-density lipoprotein or apoptotic cells induced an anti-inflammatory profile, and this required the association of PAFR with the scavenger receptor CD36 in plasma membrane lipid rafts. Blocking PAFR with selective antagonists clearly reversed the macrophages towards a pro-inflammatory profile^7^,^8^,^9^. PAFR antagonists have also been known to reverse the suppressor phenotype of tumor-associated macrophages, which resulted in the reduction of tumor growth^10^. More recently, PAF has been implicated as an important regulator of dendritic cells functions, implying its involvement not only in innate but also in adaptive immunity^11^.

Dendritic cells are a heterogeneous group of antigen presenting cells with a determinant role in the activation and suppression of immune responses, by altering the balance of the effector or regulatory T cells (Treg cells)^12^. DC properties are dependent on their maturation stage and their interaction with stimuli present in the environment. When sensing damage or pathogen associated signals, they start the maturation process, express increased levels of co-stimulatory molecules, cytokines and chemokines, and migrate into the lymphoid organs^13^. Similar to what happens in macrophages that can acquire distinct phenotypes, the exposure to anti-inflammatory cytokines and immunosuppressive agents can condition DCs to a tolerogenic state, whereas the pro-inflammatory stimuli will drive them to the activated phenotype. DCs also express PAFR^14^,^15^ and in a previous study, we found that the blockage of the PAFR in murine bone marrow-derived DCs (BM-DCs) by selective antagonists (WEB 2170, WEB 2086 and PCA 4248) during cell maturation induced by lipolysaccharide (LPS), reduced IL-10 and PGE_2_ production without affecting the IL-12. Moreover, DCs treated with PAFR-antagonists *in vitro* induced higher CD4^+^ T cell proliferation in an antigen-specific lymphocyte proliferation assay.

In this study, we suggested that the activation of PAFR in DCs, by ligands generated during LPS-induced maturation, shifts them towards a “regulatory” phenotype^11^. Since DCs are the link between innate and adaptive immune responses, we hypothesized that PAFR activation by endogenous ligands during immunization would affect the outcome of adaptive immunity. Thus, in the present study, we examined the antibody response to ovalbumin (OVA) in *wild type* (WT) mice treated with PAFR antagonist or in PAFR-deficient (PAFR-KO) mice. We also carried out an investigation to find out if PAFR antagonist treatment would increase the T cell priming activity of DCs *in vivo*. To this purpose, the immune response to OVA was transferred into *naïve* mice by injection of mature OVA-loaded BM-DCs. We found that the blockage of PAFR during immunization affected antibody production and that the transference of DCs previously treated with PAFR antagonist during maturation *in vitro* promoted an enhanced T cell response. We propose that PAF or PAF-like moieties, present at the site of immunization, have a *damping* effect on the immune response and suggest that PAFR-antagonists could be used as adjuvants in immunization protocols.

## Results

### The presence of PAFR-antagonist during immunization modulates antibodies production

**Figure 1:**
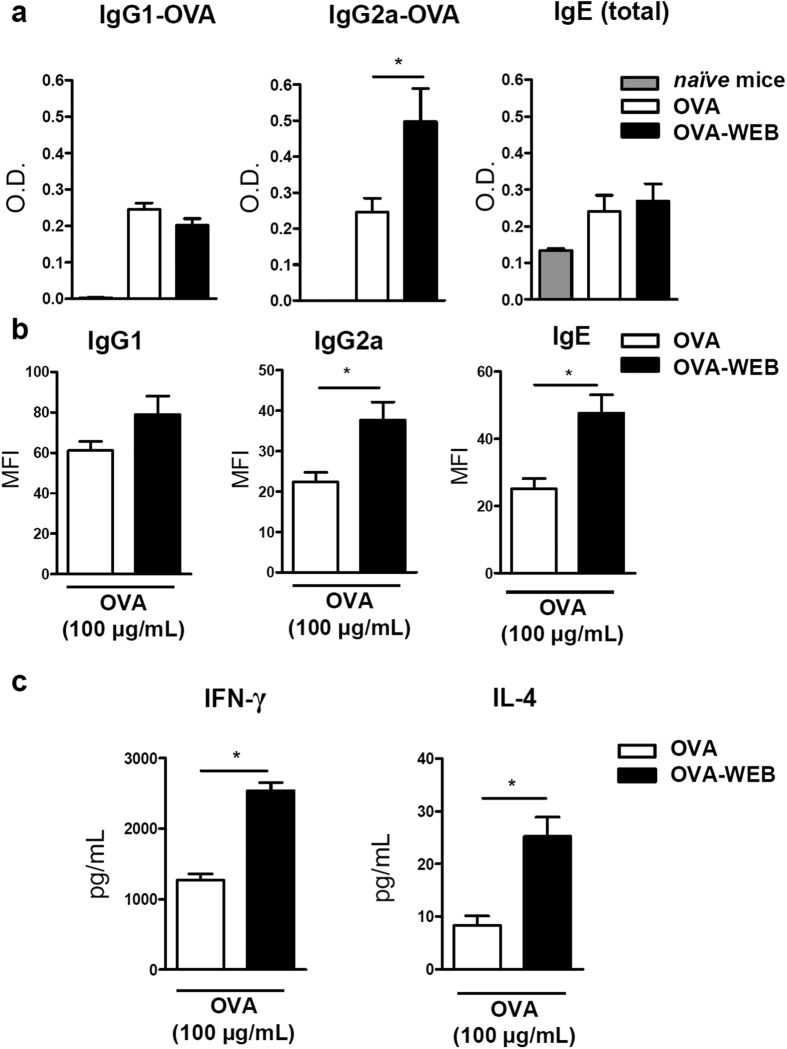
Blocking of PAFR during immunization with OVA potentiates the immune response *in vivo* and *ex vivo*. Mice were injected subcutaneously with OVA (5 μg) in saline at days 0 and 7. A group received WEB 2170 (5 mg/kg) together with the antigen. At day 14, blood was collected and IgG1 and IgG2a OVA-specific and total IgE antibodies were analyzed in the sera (sample dilutions: 1:10,000 for IgG1-OVA; 1:25 for IgG2a-OVA; and 1:100 for total IgE) (**a**). Splenocytes of immunized mice were plated (10^6^ cells/mL) and stimulated with OVA (100 μg/mL) *ex vivo.* After 72 h, supernatants were collected and total immunoglobulin isotypes were determined by a CBA assay. Positive events for individual isotypes tested were gated and values are given in MFI (“Median Fluorescence Intensity”) for each positive population observed (**b**). Cytokine (IFN-γ and IL-4) concentration in the re-stimulated splenocytes’ supernatants was assessed by ELISA (**c**). Data are representative of at least two independent experiments (*n = 5 mice/group*). *P < 0.05.

BALB/c mice were injected subcutaneously with OVA (OVA group) combined or not with the PAFR antagonist WEB 2170 (OVA-WEB group). On day 7, they were challenged with OVA or OVA plus WEB 2170 and at day 14, blood, draining lymph nodes (DLNs), and splenocytes were collected. One interesting observation was that the DLNs of OVA-WEB mice were consistently larger than those of the OVA group, and their number of total cells followed this pattern, being 8.8 ± 0.5 × 10^6^ in OVA-WEB and 5.7 ± 0.7 × 10^6^ cells in the OVA group (*P* ≤ 0.05). OVA-specific IgG1 and IgG2a antibodies were measured in the serum as surrogate markers of Th2 and Th1 responses, respectively. While IgG1-OVA specific antibody levels were similar for both groups studied, it was found that IgG2a-OVA concentration was significantly higher in OVA-WEB compared to OVA group. Total IgE was also assessed but no differences were observed between OVA or OVA-WEB groups (Fig. 1a).

Next we analyzed the *recall* response of splenocytes from both groups re-stimulated *ex vivo* with OVA (100 μg/mL). After 72 h, the supernatants of splenocytes from the OVA-WEB group presented significantly higher levels of total IgG2a and IgE than the OVA group. The IgG1 concentration was not significantly different between the groups (Fig. 1b). Moreover, higher levels of IFN-γ and IL-4 were found in the supernatants of the OVA-WEB compared to OVA group (Fig. 1c). These results indicated a modulatory effect of the PAFR antagonist on the immune response when given during immunization.

**Figure 2:**
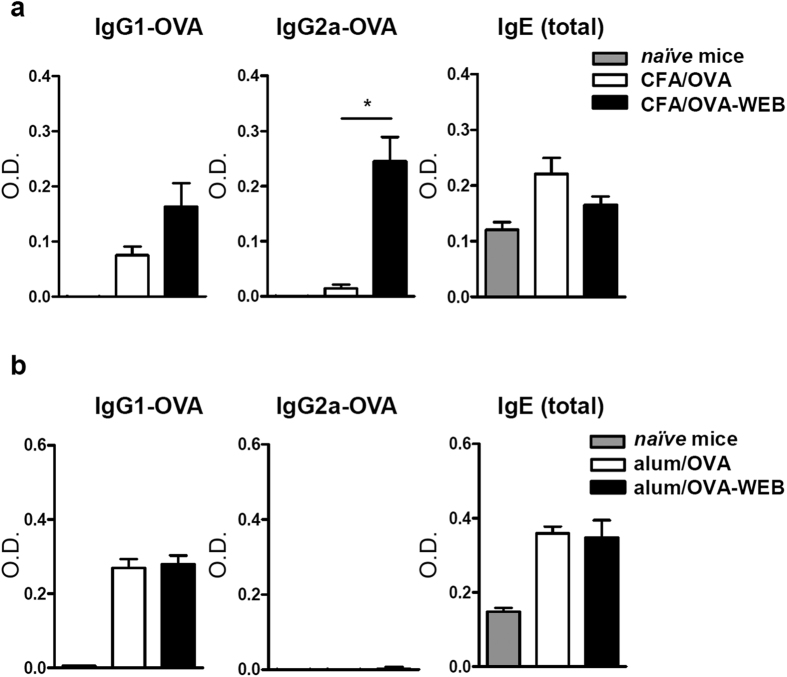
Blocking of PAFR during immunization with CFA/OVA potentiates serum levels of IgG2a OVA-specific antibody. Mice were immunized subcutaneously with OVA in complete Freund’s adjuvant (CFA) (**a**) or OVA in alum (**b**). After 7 days, they were injected once more with OVA in incomplete Freund’s adjuvant or alum, respectively. In each protocol of immunization one group received WEB 2170 (5 mg/kg) injected together with the antigen. Blood was collected at day 14 and IgG1 and IgG2a OVA-specific and total IgE antibodies were analyzed in the sera (sample dilutions: 1:10,000 for IgG1-OVA; 1:50 for IgG2a-OVA and 1:100 for total IgE). Data are representative of at least two independent experiments (*n = 5 mice/group*). *P < 0.05.

We then investigated if the PAFR antagonist would also modulate the antibody response in immunization protocols that use adjuvants. Mice were immunized with OVA in complete Freund’s adjuvant (CFA) and challenged on day 7 with OVA in incomplete Freund’s adjuvant. Figure 2a shows that the CFA/OVA-WEB group produced higher levels of antigen-specific IgG2a, whereas IgG1 serum levels did not significantly differ between the two groups. The same protocol was applied to mice immunized with OVA in alum. As expected, IgG2a OVA-specific antibody was practically undetectable (for comparative purposes the same dilutions were applied as in sera from CFA/OVA groups), and IgG1-OVA levels were not affected by the addition of the antagonist (Fig. 2b). Total IgE production was also measured in the serum of mice immunized with CFA/OVA or alum/OVA and no differences were observed when WEB 2170 was added to the immunization mixture. Overall the results suggest that ligands of PAFR are generated during immunization and that the engagement of this receptor seems to affect particularly the IgG2a isotype antibody production.

### Immune response in PAFR-deficient mice

**Figure 3:**
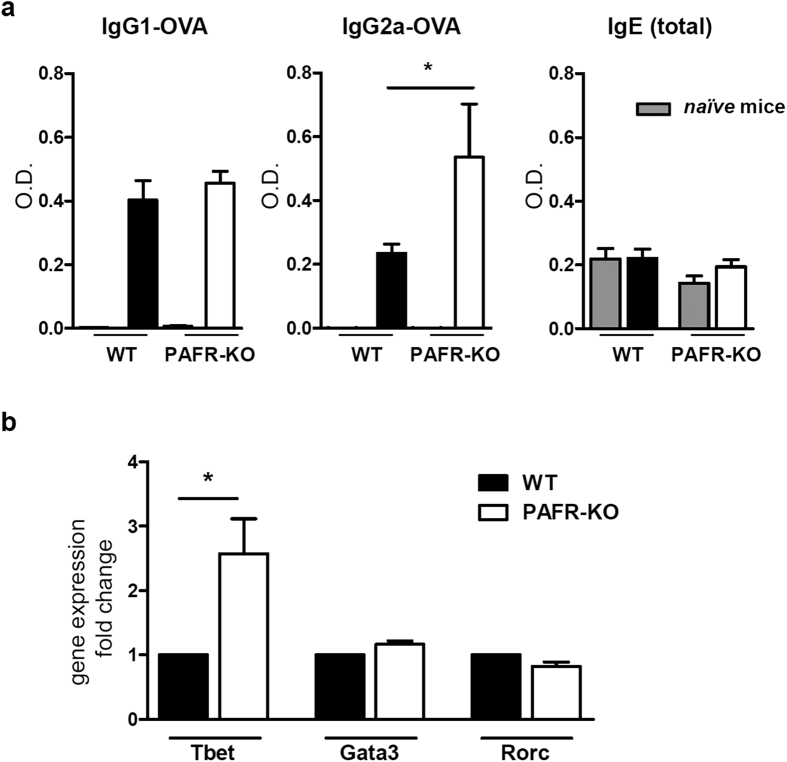
PAFR-deficient mice respond to CFA/OVA immunization similarly to wild type mice treated with PAFR antagonist. Wild type (WT) or PAFR-deficient (PAFR-KO) mice were immunized on days 0 and 7 with OVA (5 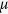
g) in complete Freund’s adjuvant CFA). Blood was collected at day 14 and IgG1 and IgG2a OVA-specific, and total IgE antibodies were analyzed in the sera (sample dilutions: 1:1,000 for IgG1-OVA, 1:25 for IgG2a-OVA and 1:100 for total IgE) (**a**). Spleens of naïve PAFR-KO and WT mice were harvested and the expression of Tbet, Gata3 and Rorc (ROR gamma) relative to internal control gene expression (Gapdh) in total splenocytes was evaluated by real-time RT-PCR. (**b**). (*n = 4–6 mice/group*). *P < 0.05.

In order to confirm this effect of PAFR activation in the immune response, we immunized PAF receptor-deficient mice (PAFR-KO) with OVA in CFA, using the same protocol described above and assessed IgG1 and IgG2a OVA-specific antibodies, and total IgE at day 14. Figure 3a shows that the Th2-related isotypes IgG1 and IgE production were similar in *wild type* (WT) and PAFR-KO mice. On the other hand, the Th1-related IgG2a was increased in the PAFR-KO mice, supporting our previous findings using PAFR antagonist.

To further understand this shifted response in the PAFR-KO mice, we performed a qPCR for Tbet, the master-regulation transcription factor for Th1 immune response, in total splenocytes of WT and PAFR-KO mice. As shown in Fig. 3b, the Tbet expression in PAFR-KO splenocytes is approximately 2.5 times higher than in WT cells. However, of the other transcription factors investigated, Gata3 and Rorc (related to Th2 and Th17 responses, respectively), were similarly expressed in both groups.

**Figure 4:**
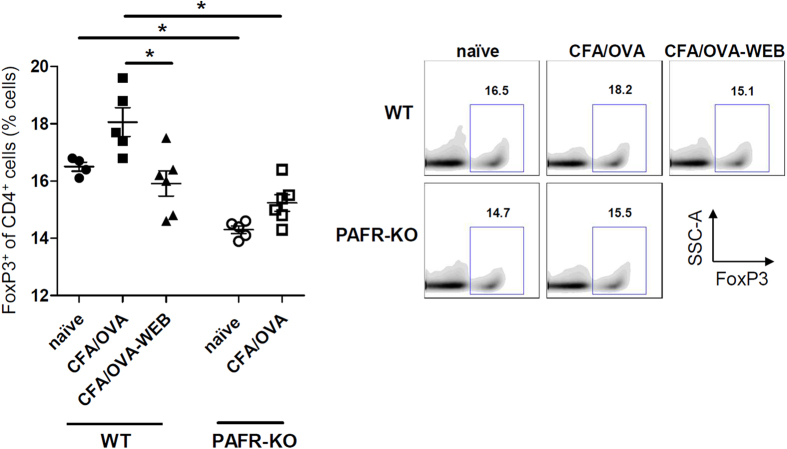
Treg cell frequency in CD4^+^ spleen cells of immunized mice is higher in PAFR-deficient mice. PAFR-KO and *wild type* (WT) mice were immunized by CFA/OVA (complete Freund’s adjuvant) and the FoxP3^+^ frequency of CD4^+^ cell population was assessed in total splenocytes of mice by flow cytometry. (*n = 4–6 mice/group*). *P < 0.05.

In the search of the T regulatory cell population (Treg cells) in the spleen of these mice, it was observed that *naïve* PAFR-KO mice had significantly less CD4^+^ T cells expressing FoxP3 than the *naïve* WT (Fig. 4). When immunized with CFA/OVA, the CD4^+^ FoxP3^+^ T cell population in the spleen increased in the WT but not in the PAFR-KO mice. When WT mice were immunized with CFA/OVA in the presence of WEB 2170, this did not occur—the percentage of FoxP3^+^ of CD4^+^ T cells was similar to the resting condition (*naïve* WT group) and to the PAFR-KO CFA/OVA group. Thus, the absence of PAFR activation during the immunization process impairs the differentiation of the Treg cell population, indicating yet a new mechanism of potentiating the immune response.

### The blockage of PAFR potentiates the T cell priming activity of DCs

In a previous study, we found that the engagement of the PAFR in DCs by endogenous PAF induced a tolerogenic phenotype, as the blockage of the receptor by antagonists during DCs maturation increased their capacity to induce CD4^+^ T cell proliferation *in vitro*^3^.

**Figure 5:**
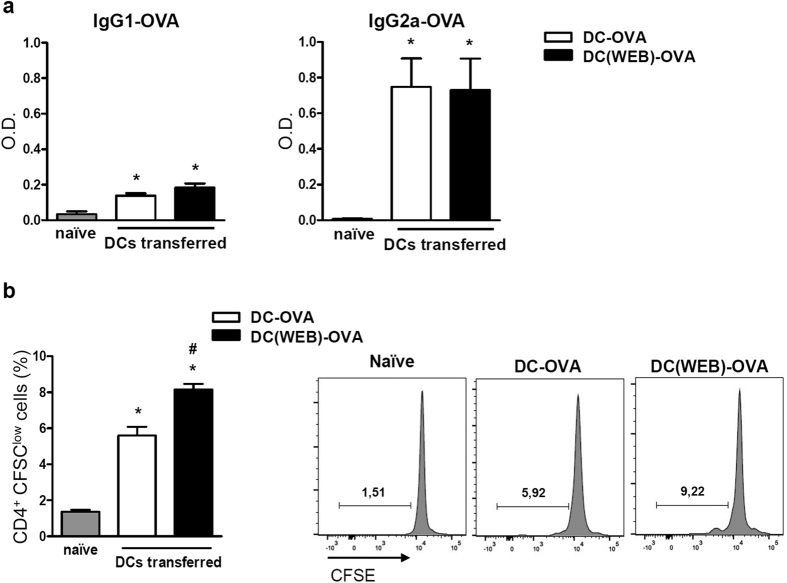
Effect of the transference of OVA-loaded BM-DCs treated with PAFR antagonist to naïve mice. BM-DCs were differentiated with GM-CSF (20 ng/mL) for 6 days. Cells were harvested, loaded with OVA (100 μg/mL), and stimulated with LPS (1 μg/mL) overnight, in the presence or not of the PAFR-antagonist (WEB 2086—50 μM). Cells were washed and re-suspended in saline. PAFR-antagonist treated DCs were transferred to a group of naïve mice via i.p. (DC-(WEB)-OVA group). Another group of mice was injected with OVA-loaded DCs without the antagonist treatment (DC-OVA group). After 7 days, the same transference protocol was performed once more. At day 14, blood was collected and IgG1 and IgG2a OVA-specific were analyzed in the serum (sample dilutions: 1:500 for IgG1-OVA and 1:10 for IgG2a-OVA) (**a**). Splenocytes of immunized mice were CFSE-labeled and co-cultured with fresh OVA-loaded BM-DCs *ex vivo* for 72 h. Proliferation of CFSE^low^ cells was assessed by flow cytometry on CD4^+^ gated cells (**b**). Data are representative of at least two independent experiments *(n = 5 mice/group)*. P < 0.05 (*vs. naïve group; ^#^*vs*. DC-OVA group).

To investigate whether the treatment with PAFR antagonist of DCs differentiated *in vitro* would boost their capacity to transfer immunity *in vivo*, bone marrow-derived DCs were loaded with OVA and treated with PAFR antagonist during maturation *in vitro*. After extensive washing to remove the antagonist, these cells were then transferred into naïve mice through two intraperitoneal injections, seven days apart. As control, a group of mice was injected with OVA-loaded DCs without the antagonist treatment. On day 14, OVA specific IgG1 and IgG2a antibodies were found in the serum of recipient mice, indicating that they were successfully immunized by DCs transference. When the transferred DCs were previously treated with the PAFR antagonist (DC(WEB)-OVA), high levels of OVA-specific IgG2a were also found, although no changes in the antibody production were observed in comparison with the DC-OVA group (Fig. 5a). Their spleens were harvested, splenocytes were incubated with fresh OVA-loaded DCs for 72 h, and CD4^+^ T cell proliferation was assessed. Under these conditions, the proliferation of CD4^+^ T cells, albeit small, was significantly higher in the group that received DCs treated with the PAFR antagonist, indicating that the blockage of PAFR in DCs during maturation improved their T cell priming activity *in vivo* (Fig. 5b). These results suggest that DCs could be one of the cells involved in the modulation of adaptive immunity by PAFR ligands.

## Discussion

Besides the well-established pro-inflammatory effects of PAF, PAFR activation in macrophages and DCs has been noted for its ability to shift these cells towards an anti-inflammatory/regulatory or tolerogenic phenotype in *in vitro* studies^7^,^8^,^9^,^11^,^16^.

In the present work, we presented evidence that the presence of a PAFR antagonist during immunization of mice with OVA alone or in emulsion with CFA potentiates the antibody response, particularly the IgG2a. This implies the generation of PAF during immunization. It is relevant to point out here that besides PAF, a wide range of oxidized phospholipids can also activate PAFR, an issue extensively reviewed by McIntyre^17^. We propose that in the inflammatory milieu at the immunization site, PAFR-ligands are generated, which act on PAFR in DCs affecting their antigen-presenting function.

In our previous study we reported that blocking of PAFR in DCs potentiated their capacity to present antigen to CD4^+^ lymphocytes in an *in vitro* assay. Here we showed that OVA-loaded DCs injected into *naïve* mice are able to transfer immunity, as shown by the presence of OVA-specific antibodies in the serum. The blockage of PAFR during DCs maturation enhanced their T cell-priming activity, assessed by T cell specific proliferation. Data presented in Fig. 5a show that, unlike when the PAFR-antagonist was given together with the antigen, when the antagonist was given to the DCs before transference to naïve mice (followed by extensive washing) it did not increase serum IgG2a-OVA concentration. Thus, blocking of PAFR only in DCs, although it potentiates T cell proliferation, does not stimulate the switch to IgG2a. In other words, activation of PAFR during immunization has a *damping* effect on the adaptive immune response, more specifically on IgG2a class switch, whereas activation of PAFR on DCs alone has the *damping* effect but no effect on the class switch.

Another intriguing finding is that the Th2-related isotypes IgG1 and IgE production were not affected by PAFR antagonists (even when alum—a strong Th2 response inducer—was added to the antigen), whereas IgG2a was clearly enhanced when mice were immunized with or without CFA. Evidence of PAF affecting antibody production is rare. There is one work showing that PAF added to B lymphoblastoid cells in culture enhanced IgG production^18^. Contrarily, another study of Influenza A virus infection reported that PAFR-KO mice produced similar levels of IgG antibodies as wild type mice^19^. Moreover, there is a short report that PAFR antagonists did not modify antigen-specific IgE production in mice immunized with alum/OVA^20^.

Our experiments with PAFR-KO mice immunized with CFA/OVA strengthened the findings obtained in the experiments where mice were treated with the PAFR antagonist. Upon immunization they produced higher levels of antigen-specific IgG2a than the WT while IgG1-OVA levels were similar between the two groups. Altogether, these data suggested that the absence of PAFR signaling during immunization potentiates antibody production favoring the IgG2 isotype production. This last assumption was supported by the augmented Tbet expression in PAFR-KO splenocytes: the Tbet transcription factor is related to Th1 cell differentiation, which controls the production of IFN-γ (the Th1 hallmark cytokine) in T cells, but its expression is also required for IgG2a IFN-γ-mediated class switch recombination in B cells^21^,^22^. IFN-γ has also been shown to induce IgG2 class-switch in B cells when acting in synergy with the Th2 cytokine IL-4, and PAF was found to boost IFN-γ production by primary lymphocytes^23^. In this work, the authors suggest that the effect of PAF to promote IgG2 switch is not directly on B cells but rather on accessory cells, which would indirectly mediate this isotype production. Nonetheless, the observation that IgG2a production requires both IFN-γ and IL-4 cytokines is in line with our results showing enhanced IgG2a-OVA antibody in the serum as well as the increased IFN-γ and IL-4 cytokines found in the *ex vivo* splenocytes re-stimulation from mice that were immunized in the presence of WEB 2170.

We also found that the Treg population (CD4^+^ FoxP3^+^ T cells) frequency in the spleen of naïve PAFR-KO mice was surprisingly lower in comparison to the WT mice. Tregs control the intensity of the immune response, and can be induced *in vivo* by adjuvants in order to restrain their inflammatory effect^24^. Indeed, in WT mice immunized with CFA/OVA, we found increased Treg frequency compared to non-immunized mice, which was reversed by PAFR antagonist. In PAFR-KO mice, immunization did not increase these cells’ frequency. Thus, the lack of PAF signaling during immunization seems to downregulate Tregs, which suggests a new mechanism for boosting immunity.

One possible way of explaining the effects hereby described is to assume that the inflammation caused by the antigen injection, particularly in combination with an adjuvant that bears pathogen-associated molecular patterns such as CFA, triggers cells present in the inoculation site to produce PAF or PAF-like molecules that activate PAFR expressed in innate immune cells, thus affecting the outcome of adaptive immune response. It has been shown that cellular damage generates a series of phospholipids that bind to PAFR, particularly UV exposure, which promotes systemic immune suppression^25^. We can speculate that the presence of a PAFR antagonist at that moment would abrogate the “damping effect” of PAF on innate immune cells, among them the DCs, and consequently, their T cell priming function would be enhanced. How this will determine which type of helper T cell will be affected remains to be determined. In addition, we cannot rule out the possibility of the antagonist or agonist of PAF being drained to the lymph nodes and acting directly on the B cells or follicular DCs (FDCs). The fact that both B cells and FDCs express PAFR and PAF has been described as having an immunomodulatory role in B cells and being important to B cell-FDCs interactions^26^,^27^, this possibility should be acknowledged.

Collectively, our results indicate a new function of PAFR-agonists as molecules that can fine tune not only innate, but adaptive responses as well, suggesting a potential application of PAFR antagonists in DCs-based vaccination protocols for cancer and other diseases, or the use of a PAFR antagonist as an adjuvant in immunization protocols.

## Methods

### Mice

Eight to ten-week old BALB/c male wild type (WT) or PAFR-deficient (PAFR-KO) mice were obtained from the Department of Immunology’s Animal Facility at the University of São Paulo, and were housed under specific pathogen-free conditions. Experiments were performed following the guidelines for animal use and care approved by the Institutional Animal Care and Use Committee of the Institute of Biomedical Sciences at the University of São Paulo. PAFR-deficient mice were originally provided by Dr. Satoshi Ishii and Dr. Takao Shimizu^28^.

### Immunization protocol

Mice were anesthetized by intraperitoneal injection of ketamine (100 mg/kg)/xylazine (10 mg/kg), and were immunized subcutaneously in the flanks of the lower back, with 5 μg of chicken ovalbumin (OVA grade V; Sigma-Aldrich, Saint Louis, MO, USA), according to the following protocols: a) OVA in saline (200 μL); b) OVA in 100 μL of complete Freund’s adjuvant (CFA, Sigma-Aldrich) and 100 μL saline or c) OVA in 30 μL of aluminium hydroxide gel (alum, Reheis, Berkeley Heights, NJ, USA), and 170 μL saline. At day 7, mice were boosted by the subcutaneous injection of OVA in saline, OVA in incomplete’s Freund adjuvant (Sigma-Aldrich), or OVA in alum, respectively. In each protocol, a group of mice received OVA together with the PAFR antagonist WEB 2170 (5 mg/kg; Boehringer Ingelheim, Pharma KB, Biberach, Germany)^29^, on days 0 and 7. On the 14^th^ day, blood, spleen, and the inguinal draining lymph nodes were collected. After blood collection, samples were left at 4 °C for 30 min, followed by centrifugation (2,000 × *g* for 10 min in a refrigerated centrifuge). Serum samples were transferred to new tubes and stored at −80 °C.

### Bone marrow-derived dendritic cells (BM-DC) generation and transference

Bone marrow cells were collected from the femurs of BALB/c mice and cultured in RPMI 1640 (GIBCO, Grand Island, NY, USA) medium, supplemented with 10% heat-inactivated fetal bovine serum (FBS, GIBCO, GO, Brazil), and antibiotic-antimycotic (GIBCO, Grand Island, NY, USA). BM-DCs differentiation was induced by 20 ng/mL of granulocyte–macrophage colony-stimulating factor (rmGM-CSF, Peprotech, Rocky Hill, NJ, USA). After 6 days, the cells were harvested, pulsed with 100 μg/mL of chicken ovalbumin (OVA grade V; Sigma-Aldrich, Saint Louis, MO, USA), and treated with 50 μM of the PAFR antagonist WEB 2086^30^ (Tocris Biosciences, Ellisville, MO, USA), for 30 min. LPS (1 μg/mL, Sigma-Aldrich), was then added to induce cell maturation. After 24 h, the cells were extensively washed to remove free OVA, and were re-suspended in saline at 10^6^ cells/100 μL. Three to five million cells were transferred to naïve mice via intraperitoneal injection. This process was repeated after 7 days. At day 14 of the first cell transference, the mice were euthanized by CO_2_ exposure, and their serum and spleen were collected.

### *Ex vivo* cultures and cell analysis

Spleen and draining lymph nodes were collected and passed through a 70 μm nylon cell strainer to remove connective tissues. The cells obtained were incubated with ACK Lysis Buffer (GIBCO), to lyse the red blood cells. For *ex vivo* stimulation, splenocytes were plated at 10^6^ cells/mL, and incubated with 100 μg/mL of OVA. Supernatants were collected after 72 h and stored at −80 °C until use. For proliferation assay, the splenocytes were labeled with CFSE (CellTrace CFSE Cell Proliferation kit, Invitrogen, Eugene, OR, USA), and co-cultured with fresh OVA-loaded BM-DCs generated as described above in a 4:1 ratio (splenocytes:BM-DCs) for 72 h. After that, cells were stained at 4 °C for 30 min with anti-mouse CD4-APC (L3T4) (BD Pharmingen, San Jose, CA, USA) antibody. For Treg detection, splenocytes were stained with the Mouse Regulatory T Cell Staining Kit #1 (Affymetrix eBiosciences, San Diego, CA, USA) according to the manufacturer’s instructions. Data was acquired using a FACSCanto II flow cytometer (BD Biosciences), and analyzed by the FlowJo software (TreeStar, Ashland, OR, USA). For both proliferation and Treg detection assays, doublets were excluded, and analyses were conducted on CD4^+^ gated cells.

### Total antibody and cytokines determination

Supernatants of *ex vivo* stimulated cells were collected and the total antibody profile was determined by a Cytometric Bead Array (CBA, Mouse Immunoglobulin Isotyping Kit; BD Biosciences), using a FACSCanto II Flow Cytometer. Data were analyzed by FlowJo software. Positive events for each isotype tested were gated and analyzed as Median Fluorescence Intensity (MFI) in a semi-quantitative way (higher MFI meaning higher amount of antibody detected).

IL-4 and IFN-γ were assayed by OptEIA ELISA (BD Biosciences). Absorbance was evaluated at 450 nM in the microplate reader SpectraMax 190 and was analyzed by SoftMax Pro 4.0 software (both from Molecular Devices, Sunnyvale, CA, USA). Values were expressed as pg/mL, which were deduced from standard curves of recombinant cytokines run in parallel.

### Determination of OVA-specific IgG1 and IgG2a

To determine OVA-specific IgG1 and IgG2a antibodies, plates were coated overnight at 4 °C with OVA (20 μg/mL) in phosphate-buffered saline (PBS). Plates were washed three times with wash buffer (PBS containing 0.05% Tween-20), and blocked with assay diluent (PBS containing 10% heat-inactivated fetal bovine serum) for 1 h. Mice serum samples were diluted in assay diluent and added to plates after three more washes. Following a 2 h incubation at room temperature (RT), plates were washed five times, and anti-IgG1 (BD Pharmingen) or anti-IgG2a (Invitrogen) conjugated with peroxidase were used as secondary antibodies. Plates were incubated for 1 h RT and washed seven times. TMB Substrate Reagent Set (BD Biosciences) was added and plates were left for 30 min RT in the dark. Colorimetric reaction was stopped by the addition of 2 NH_2_SO_4_. Absorbance was acquired at 450 nM in the microplate reader SpectraMax 190 and was analyzed by SoftMax Pro 4.0 software. Absorbance sample values were considered after subtracting values of wells incubated with fresh serum from naïve mice in the same dilutions or incubated with assay diluent. Absorbance values for diluents were close to those obtained with control serum at the dilution used for immune serum. Data are shown in optical density (OD) units.

### Real-time RT-PCR

Spleens were collected and passed through a 70 μm nylon cell strainer to remove connective tissue, and red blood cells were lysed with ACK Lysis Buffer (GIBCO). Total RNA was isolated using the Trizol Reagent (Invitrogen, Carlsbad, CA, USA), according to the manufacturer’s instructions. cDNA was generated from the total RNA, using the RevertAid First Strand cDNA Synthesis Kit (Thermo Scientific Fermentas, Vilnius, Lithuania). Real-time PCR was performed with the StepOnePlus Real-Time PCR System (Applied Biosystems, USA), using SYBR Green (Fast SYBR Green Master Mix, Applied Biosystems, Warrington, UK), and specific primers for Tbet (forward, 5′-CGC TCA CTG CTC GGA ACT CT-3′ and reverse, 5′-TCC TGT CTC CAG CCG TTT CT-3′), Gata3 (forward, 5′-GCC TGT GCA AAA GAG ATT TCA GAT-3′ and reverse, 5′-TGA TTC ACA GAG CAT GTA GGC C-3′), Rorc (Mm.PT.58, 8455991–IDT, Coralville, IA, USA) and Gapdh (forward, 5′–AGG TCG GTG TGA ACG GAT TTG–3′ and reverse, 5′–TGT AGA CCA TGT AGT TGA GGT CA–3′). Relative gene expression was calculated using the 2^−ΔΔCT^ method as previously described^31^. Data were shown in the fold change expression relative to the internal control gene (Gapdh).

### Statistical analysis

Results were presented as mean values ± SEM. Statistical differences between mean values were determined by the one-way ANOVA, followed by the Newman-Keuls test or Student’s *t* test, using GraphPad Prism 5.0 software (San Diego, CA). P < 0.05 values were considered as significant.

## Additional Information

**How to cite this article**: Koga, M. M. *et al*. Boosting Adaptive Immunity: A New Role for PAFR Antagonists. *Sci. Rep.* **6**, 39146; doi: 10.1038/srep39146 (2016).

**Publisher's note:** Springer Nature remains neutral with regard to jurisdictional claims in published maps and institutional affiliations.
